# Classification of Tea Quality Levels Using Near-Infrared Spectroscopy Based on CLPSO-SVM

**DOI:** 10.3390/foods11111658

**Published:** 2022-06-05

**Authors:** Yuhan Ding, Yuli Yan, Jun Li, Xu Chen, Hui Jiang

**Affiliations:** 1 Key Laboratory of Modern Agricultural Equipment and Technology, Jiangsu University, Ministry of Education, Zhenjiang 212013, China; yhding@ujs.edu.cn (Y.D.); j.li@seu.edu.cn (J.L.); 2 High-Tech Key Laboratory of Agricultural Equipment and Intelligence of Jiangsu Province, Jiangsu University, Zhenjiang 212013, China; 3 Institute of High-Performance Electrical Machine System and Intelligent Control, Jiangsu University, Zhenjiang 212013, China; 4 School of Electrical and Information Engineering, Jiangsu University, Zhenjiang 212013, China; yanyuli2325@126.com (Y.Y.); xuchen@ujs.edu.cn (X.C.); 5 School of Automation, Southeast University, Nanjing 210096, China

**Keywords:** Huangshan Maofeng tea, near-infrared spectroscopy, tea quality level, classification, CLPSO-SVM

## Abstract

In this paper, we propose a method for classifying tea quality levels based on near-infrared spectroscopy. Firstly, the absorbance spectra of Huangshan Maofeng tea samples were obtained in a wavenumber range of 10,000~4000 cm^−1^ using near-infrared spectroscopy. The spectral data were then converted to transmittance and smoothed using the Savitzky–Golay (SG) algorithm. The denoised transmittance spectra were dimensionally reduced using principal component analysis (PCA). The characteristic variables obtained using PCA were used as the input variables and the tea level was used as the output to establish a support vector machine (SVM) classification model. The penalty factor *c* and the kernel function parameter *g* in the SVM model were optimized using particle swarm optimization (PSO) and comprehensive-learning particle swarm optimization (CLPSO) algorithms. The final experimental results show that the CLPSO-SVM method had the best classification performance, and the classification accuracy reached 99.17%.

## 1. Introduction

Tea level or tea grade is a comprehensive evaluation of tea quality and is related to many factors, such as the age of the tea leaves, the color of the produced tea, and the aroma and taste of the tea soup. In terms of composition, the tea level is mainly related to the various chemical substances it contains. For example, the content of cellulose and hemicellulose can determine the age and tenderness of the tea leaves, and the content and proportion of amino acids, tea polyphenols, and caffeine can determine the nutrition, and also the taste of the tea. However, many other factors affect the tea levels [1,2]. Because of the complexity and subtlety of tea level standards, the boundaries of tea levels are not clear, which gives profiteers the opportunity to cheat customers with low-level teas at high prices. Therefore, it is of great significance to find a reliable, fast, and simple method for identifying tea levels.

As a simple, rapid, and non-destructive detection method, near-infrared (NIR) spectroscopy has been successfully applied to the quality analysis of various agricultural products such as tobacco, coffee, and other crops, with good results [3,4,5,6,7,8,9].

For tea classification, NIR spectroscopy also has certain applications. In 2006, Zhao et al. used a support vector machine (SVM) and back-propagation artificial neural network (BP-ANN) methods to quickly identify black tea, green tea, and oolong tea, based on NIR spectroscopy technology [10]. The results show that SVMs could quickly and effectively identify tea types. In 2018, Anindya et al. used NIR spectroscopy and principal component analysis (PCA) to quickly and non-destructively classify Indonesian black tea. The results show that this method can be used as a potential method to identify the tea grade [11]. In 2019, Li et al. used a specific spectral region for synergy interval partial least square (siPLS) modeling based on NIR spectroscopy technology, and obtained a 97% and 93% prediction accuracy rate of extra-flat tea in cross-validation and external verification, respectively [12]. Then, based on NIR spectroscopy, Firmani et al. combined partial least square discriminant analysis (PLS-DA) and SIMCA software to identify Darjeeling black tea [13]. In 2020, Li et al. used SVMs with particle swarm optimization (PSO) to identify fresh-leaf white tea of different maturity levels based on NIR spectroscopy technology, and achieved significant results [14].

Classification methods are commonly established using PLS-DA [15], extreme learning machines (ELMs) [16], support vector machines (SVMs) [17], decision trees [18], artificial neural networks [19], etc. SVMs have particularly low generalization error rates and are suitable for small data sets [20,21].

In order to improve the accuracy of SVM models, some scholars have combined intelligence optimization methods with the traditional SVM methods and have applied them in a wide range of fields [22,23,24,25]. Intelligent optimization algorithms mainly include the genetic algorithm (GA) [26], clonal algorithm (CA) [27], differential evolution (DE) algorithm [28], particle swarm optimization (PSO) algorithm [29], etc. Among them, PSO is widely used because of its fast convergence and easy implementation [30]. Although the convergence speed of PSO is fast, it has the problems of premature convergence, local optimization, and low efficiency of later iterations.

To overcome these problems, we applied a comprehensive-learning particle swarm optimization (CLPSO) algorithm to optimize the SVM model to classify six levels of Huangshan Maofeng tea [31,32]. The CLPSO algorithm can prevent particles from undergoing premature convergence and avoid them falling into a local optimum, offering the algorithm good robustness; thus, it has better optimizing effects and is promising for making the optimized SVM model obtain better classification results.

## 2. Materials and Methods

### 2.1. Tea Sample Preparation and Spectral Acquisition

The experimental Huangshan Maofeng tea samples were provided by Xie Yuda Tea Co., Ltd, Huangshan, China. They are produced and labeled to six different levels, strictly according to the national standard GB/T 19460-2008. In our experiments, six levels of tea (20 samples of each level) were weighed using a Shimadzu electronic balance AUY120, with each sample being 3 g ± 0.005 g.

The NIR spectroscopy analyzer used for the acquisition of the spectra was an Antaris II Fourier-transform near-infrared spectrometer from Thermo Fisher Scientific, Madison, WI, USA.

**Figure 1:**
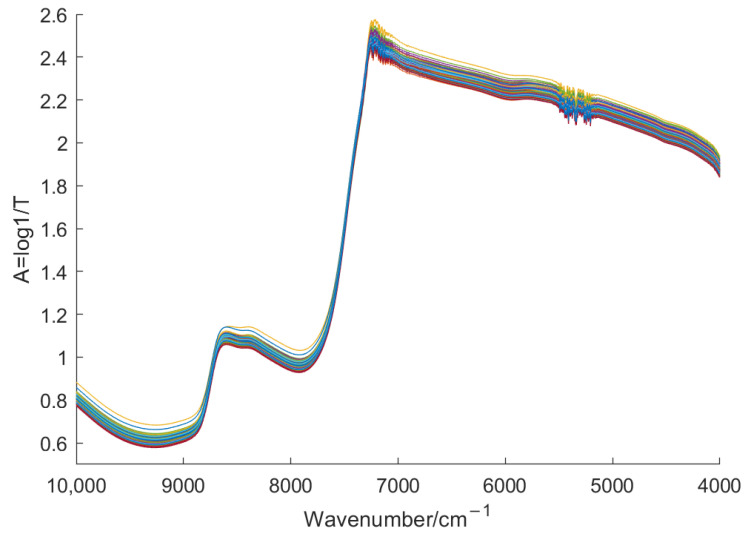
Original spectra of Huangshan Maofeng tea.

During the experiments, the laboratory temperature was kept at 20 °C and the humidity was constant. First, 150 mL of 100 °C boiling water was measured using a measuring cylinder and poured into an Erlenmeyer flask with one tea sample. The tea leaves were brewed in the boiling water for 5 min, and the tea residue was filtered out using filter paper. Part of the tea soup was then moved into a transparent cuboid quartz container using a pipette, as the scanning sample. Finally, the sample was scanned using the NIR spectrum analyzer in transmittance mode, with a scanning wavenumber range of 10,000~4000 cm^−1^ and a resolution of 3.9 cm^−1^. Each sample was scanned 3 times in this way, and its average spectrum was taken as the original spectrum of the sample [33], as shown in Figure 1.

### 2.2. Data Preprocessing

**Figure 2:**
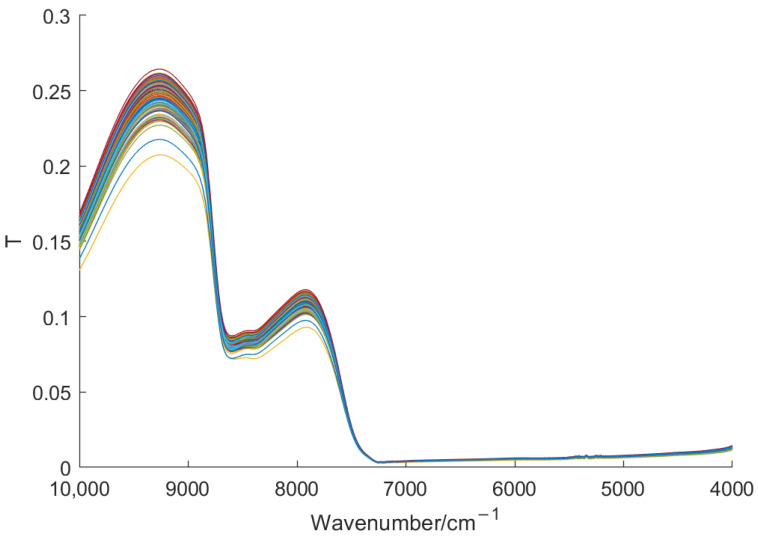
Transmittance spectra of Huangshan Maofeng tea.

The original spectra of the tea sample in Figure 1 are absorbance spectra. From Figure 1, it can be seen that there exists strong absorbance in the wavenumber below 7500 cm^−1^, and there is no effective information there. Directly using such spectra to construct a classification model would make the model ill-conditioned and affect its performance. Therefore, we converted the absorbance spectra to transmittance ones, as shown in Figure 2.

Due to the inevitable measuring error, noise, and redundant information generated during the measurement of the NIR instrument, the Savitzky–Golay (SG) filtering method (with a frame length of 21) was used to smooth the spectral data and reduce the noise interference [34].

Since there were 1557 data points in each sample, principal component analysis (PCA) was performed on the data to reduce the dimensions [35]. The 1557 dimensions of the feature were mapped to the *k* dimensions (*k* < *n*), which could effectively reduce the dimension and operation, maximize the difference in the retained data from the perspective of variance, and avoid over-fitting.

**Figure 3:**
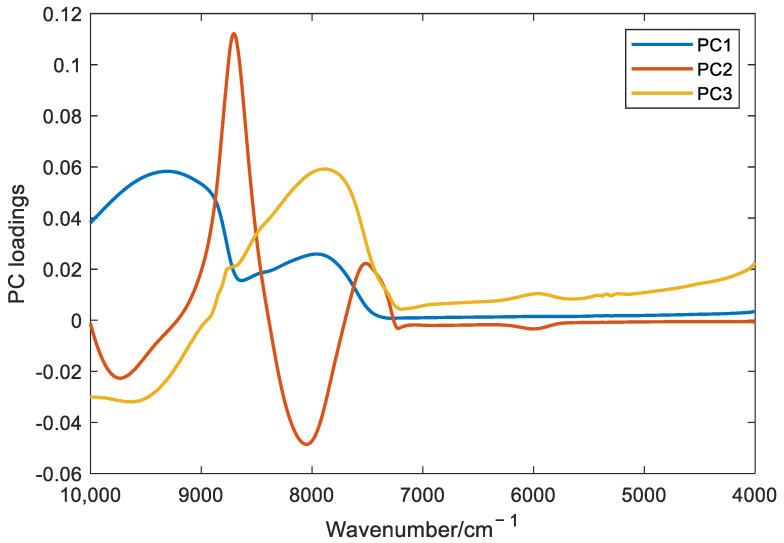
PC loadings of filtered transmittance spectral data.

Figure 3 is the loading plot of the first 3 principal components (PCs). From Figure 3, it can be seen that for PC 1, which can explain 91.8% of the variance, the spectral contribution from wavenumbers below 7500 cm^−1^ is very close to zero; for PC 2, whose variance explanation is 4.1%, the spectral contribution below 7250 cm^−1^ is almost zero; and for PC 3, which can only explain 2.7% of the variance, the spectral contribution below 7250 cm^−1^ is also close to zero, and the relatively large contribution only appears below 5000 cm^−1^, where the transmittance spectra are also relatively large. The model, when established by such principal components, has a small proportion of the input with low transmittance, so the model, when established in this way, will possess high reliability.

### 2.3. SVM Model

In this paper, we used SVMs to establish the tea level classification model. SVMs search for a hyperplane to segment samples according to the positive and negative classes [36]. In the sample space, the division of the hyperplane can be described using Equation (1):

(1) $$y(x)=\omega_{1}^{T}\phi (x)+b$$

In Equation (1), *ω*_1_ is the normal vector of the hyperplane, *x* is the sample data, and *b* is the offset. Based on the principle of minimum structural risk, the SVM avoids learning problems and has a strong generalization ability. It converts the solving problem into a convex quadratic programming problem with linear constraints. The problem can be described using Equations (2)–(4):

(2) $$\min\frac{1}{2}∥\omega_{1}{∥}^{2}+c\overset{n}{\underset{i=1}{\sum }}\xi_{i}$$

(3) $$\xi_{i}\geq 0(i=1,2,\cdots n)$$

(4) $$K\left(x_{i},x\right)=\exp\left(-\frac{∥x_{i}-x{∥}_{2}^{2}}{2g^{2}}\right)$$

where *ξ* is the relaxation factor; *K*(*x_i_*, *x*) is the kernel function of the SVM model; *c* is the penalty parameter, which determines the model’s tolerance for wrong samples; and *g* is the kernel function parameter, which determines the form of the classification hyperplane. The purpose of the parameter selection of the SVM model is to adjust the values of *c* and *g* within certain ranges, to give the SVM model better classification accuracy.

In practical application, the problems to be solved are multi-classification ones, and an SVM is a two-category classification model, so the model of multi-SVMs is used as well to deal with the multi-classification problems. In this research, we constructed six SVMs during the training. The first SVM can discriminate Level 1 samples and other samples by assigning Level 1 samples positive scores and other samples negative scores. The second SVM can discriminate Level 2 samples and other samples, and Level 2 samples will obtain positive scores while other samples will obtain negative scores. Similar work is performed on the third-to-sixth SVMs. When a test sample is input to the six SVMs, we obtain six different scores, and the ultimate classification result of the sample is determined according to the largest score.

### 2.4. PSO Algorithm

Suitable values of *c* and *g* are helpful to improve the accuracy of an SVM classification model. The purpose to the particle swarm optimization (PSO) algorithm is to find these suitable values using the training samples.The PSO algorithm has the characteristics of fast convergence, good solution quality, and good robustness for multidimensional spatial functions or dynamic target problems [37]. In the PSO algorithm, the position of each particle contains a pair of SVM parameters, *c* and *g*, and the purpose of the procedure for optimization is to find the most suitable position that maximizes the classification accuracy of the training samples. During the iteration process, the particles track the individual extremum (*P_id_*, the best position of a particle) and the global extremum (*P_gd_*, the best position of all particles) and then update the speed *V_id_* and position *X_id_* of the current particle according to Equations (5) and (6):

(5) $$V_{id}^{\left(k+1\right)}=V_{id}^{\left(k\right)}+c_{1}r_{1}\left(P_{id}^{\left(k\right)}-X_{id}^{\left(k\right)}\right)+c_{2}r_{2}\left(P_{gd}^{\left(k\right)}-X_{id}^{\left(k\right)}\right)$$

(6) $$X_{id}^{\left(k+1\right)}=X_{id}^{\left(k\right)}+V_{id}^{\left(k\right)}$$

In Equations (5) and (6), *i* ∈ *N* (*N* represents the total number of particles), *d* represents the dimension, *k* and *k* + 1 represent the numbers of current and next iterations, and *c*_1_ and *c*_2_ are the acceleration factors.

All the particles in the PSO algorithm are the possible solution to *c* and *g*, and the best particle when iteration ends will be used as the best *c* and *g* to establish the optimized SVM classification model, to obtain better accuracy.

### 2.5. CLPSO Algorithm

In the basic PSO, the convergence speed of the algorithm is fast, but the local search ability is not strong, which directly leads to low search accuracy and, often, local convergence. In order to solve the above problems, comprehensive-learning particle swarm optimization (CLPSO) is proposed [38,39,40]. In CLPSO, each particle in the population is able to obtain the best learning experience from different dimensions of other particles, rather than following the best individual alone. This is the essential difference between CLPSO and basic PSO. The iterative formula is as shown in Equations (7)–(9):

(7) $$V_{id}^{\left(k+1\right)}=\omega^{\left(k\right)}V_{id}^{\left(k\right)}+c_{a}r_{id}\left(p_{best}^{\left(k\right)}-X_{id}^{\left(k\right)}\right)$$

(8) $$X_{id}^{\left(k+1\right)}=X_{id}^{\left(k\right)}+V_{id}^{\left(k\right)}$$

(9) $$f_{i}\left(d\right)=\left[f_{i}\left(1\right),f_{i}\left(2\right),\ldots ,f_{i}\left(D\right)\right]$$

In Equation (7), *c_a_* represents the acceleration factor (*c_a_* = 1.49445). In Equation (9), $f_{i}\left(d\right)$ means that particle *i* learns from the individual extremum *pbest* of a particle in the *d* dimension, which is determined by the learning probability $Pc_{i}$. $Pc_{i}$ is calculated using Equation (10):

(10) $$Pc_{i}=a+\frac{b\left(e^{10\left(i-1\right)/\left(pop-1\right)}-1\right)}{e^{10}-1}$$

where *pop* is the population size (*pop* = 40), and *a* and *b* are two parameters for determining the maximum and minimum learning probabilities (*a* = 0, *b* = 0.5).

As can be seen from Equations (5) and (7), another difference between the two methods is that CLPSO introduces inertia weight (*ω*^(*k*)^), which reflects the ability of a particle to inherit the previous velocity of the particle. Studies show that a larger weight is more beneficial to the global search, while a smaller weight is more beneficial to the local search [40]. In order to better balance the global search and local search ability of the algorithm, a linear decreasing inertia weight is proposed, as shown in Equation (11):

(11) $$\omega^{\left(k\right)}=\omega_{\max}-\frac{\left(\omega_{\max}-\omega_{\min})(\max FEX-k\right)}{\left(\max FEX\right)}$$

where max*FEX* represents the maximum number of iterations (max*FEX* = 40), and *ω*_max_ and *ω*_min_ are the initial inertia weight and the ending inertia weight (*ω*_max_ = 0.9, *ω*_min_ = 0.4). With the increase in iterations, the inertia weight linearly decreases from 0.9 to 0.4, which realizes the global search in the initial iterations and the local exact search in the later iterations, and controls the convergence speed effectively.

If the penalty factor *c* and the kernel function parameter *g* in the SVM model are optimized by the CLPSO algorithm, the iterative steps are as follows:

Step 1: Establish an SVM model and initialize the SVM parameter combination (*c*, *g*) randomly.

Step 2: For each particle of CLPSO (the particle represents *c* and *g*, which can possibly solve the problem), initialize its velocity and position and set the inertia weight randomly.

Step 3: Calculate the current fitness value (*fit*(*X_id_*), the classification accuracy of training samples), and store the individual extreme value (*pbest*) of the optimal position.

Step 4: Update the velocity and position of the particles according to Equations (7)–(9).

Step 5: Calculate the fitness value of the new position and update the *pbest*.

Step 6: End the algorithm if the iteration number reaches the maximum and substitute the optimized (*c*, *g*) into the SVM model; otherwise, go to Step 4.

### 2.6. Software

All the algorithms and the statistical analysis were implemented in Matlab R2021a (Mathworks, Natick, MA, USA) under Windows 10 in data processing.

## 3. Results and Discussion

Based on the NIR spectroscopy technique and the experimental method proposed in Section 2, six levels of Huangshan Maofeng tea were classified and identified.

In the testing experiments, we randomly divided the 120 samples into four groups, each with 30 samples. Each time one group of samples was selected as the testing sample, the other three groups were used as training samples to construct a classification model. By repeating this four times, every sample was tested. The final testing accuracy was calculated from the average of the four tests.

We first conducted experiments for tea level classification based on the basic SVM models, with *c* = 2, *g* = 1. Different characteristic variables obtained by PCA were used as the input variables of the classification models. The tea levels are the output variables. The tea level is expressed by a number from 1 to 6. Number 1 represents level 1, number 2 represents level 2, etc.

**Figure 4:**
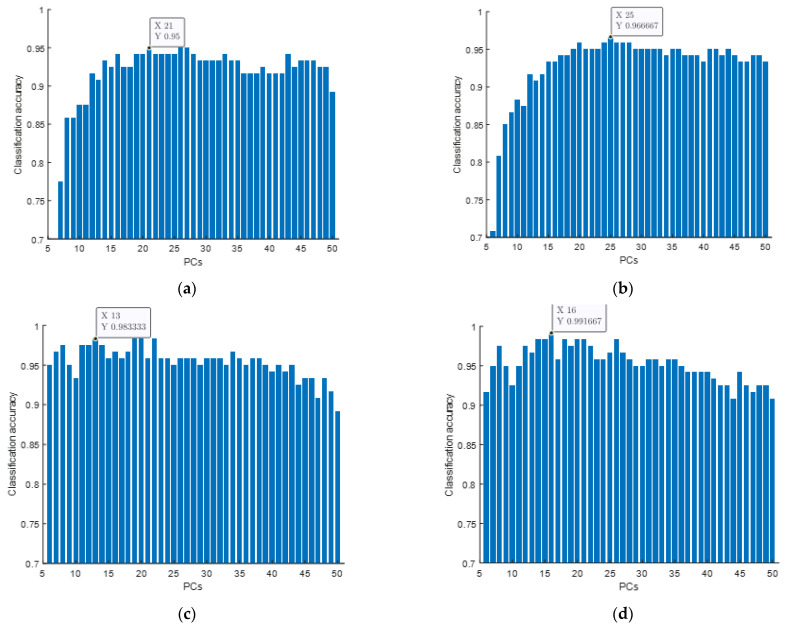
Testing results obtained using different models: (**a**) results from SVM; (**b**) results from PLS-DA; (**c**) results from PSO-SVM; (**d**) results from CLPSO-SVM.

Figure 4a shows the classification results obtained using basic SVM models. In the figure, the x-axis represents the number of PCs used by the model as input variables, and the y-axis represents the average testing accuracy for different samples. From the figure, we can see that basic SVM models can virtually realize the tea level classification. The best SVM model can obtain an accuracy of 95% when the input number is 21.

As a reference, we also gave the classification results of the traditional PLS-DA model, as shown in Figure 4b. From this figure, it can be seen that the accuracy of the PLS-DA models is a little higher and a little steadier than that of the basic SVM models. However, it should be noted that the PLS-DA model has no adjustable parameters. Its classification effect is completely determined by the training samples, meaning it lacks flexibility, while the classification performance of the SVM model is determined by the penalty factor *c*, the kernel function parameter *g*, and the training samples together. Therefore, through the adjustment of *c* and *g* parameters, the SVM model can be optimized and can be better adapted to different research objectives.

In this research, *c* and *g* were optimized by PSO and CLPSO. The optimized *c* and *g* were then used in the optimized SVM models to classify the tea levels. The classification accuracies of the PSO-SVM models and CLPSO-SVM models are as shown in Figure 4c and Figure 4d, respectively. All the key numbers are also listed in Table 1.

As can be seen from Figure 4 and Table 1, the accuracy of the best PSO-SVM model on the training set is 100%, and the accuracy on the testing set reaches 98.33%. However, the input number is also reduced to 13.

Furthermore, since the CLPSO algorithm effectively controls the convergence speed, and realizes the global search in the initial iteration and the local exact search in the later iteration, the CLPSO-SVM method has the best classification results. The classification accuracy of the training set is also 100%, and the classification accuracy of the testing set is 99.17%, with the input number being 16.

**Figure 5:**
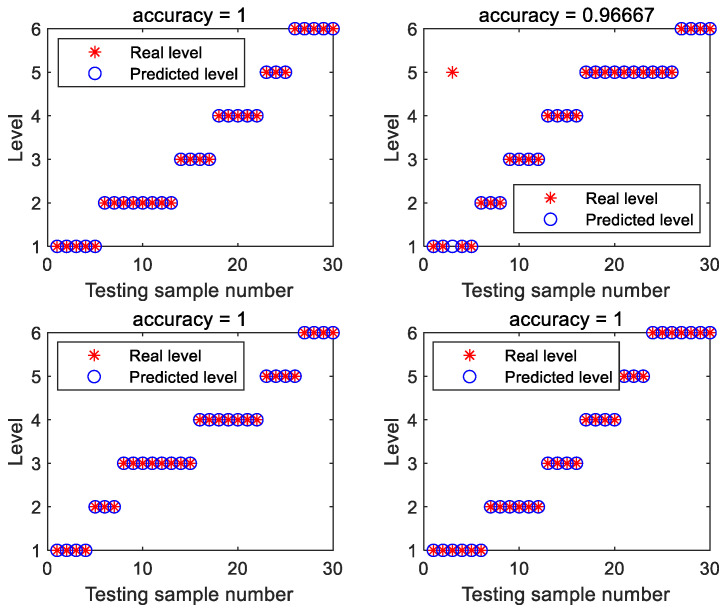
Detailed testing results obtained using the CLPSO-SVM model, with the PC number being 16.

Figure 5 shows the detailed testing results obtained by the best CLPSO-SVM model, with PC number being 16. From the figure, it can be seen that almost all the tea samples were classified correctly, and only one sample in one testing set was misclassified.

## 4. Conclusions

In this study, based on the collected NIR spectral data of Huangshan Maofeng tea, classification models were established using SVMs. In order to improve the classification accuracy, the penalty factor *c* and the kernel function parameter *g* in the SVM models were optimized using PSO and CLPSO algorithms. Comparing the classification results obtained using the basic SVM model and the models optimized using PSO and CLPSO, it can be concluded that:

(1) The basic SVM model based on NIR spectral data can virtually realize the tea level classification. Though its classification accuracy is not as high as the traditional PLS-DA model’s, it has the potential to be optimized.

(2) PSO and CLPSO algorithms can effectively improve classification accuracy by optimizing the SVM parameters.

(3) The CLPSO-SVM model achieved the highest classification accuracy among all four models. It obtained a classification accuracy of 99.17% in the testing samples, and only one sample was misclassified. It is a reliable method and has broad prospects in tea -classification applications.

## Figures and Tables

**Table 1:** Comparison of four methods.

Method	PCs	Training Accuracy	Testing Accuracy
SVM	21	99.17%	95%
PLS-DA	25	99.17%	96.67%
PSO-SVM	13	100%	98.33%
CLPSO-SVM	16	100%	99.17%

## Data Availability

The data that support the findings of this study are available upon request from the corresponding author. The data are not publicly available due to privacy or ethical restrictions.
